# Relationships, Decisions, and Physical Effort in the Marro Traditional Sporting Game: A Multimodal Approach

**DOI:** 10.3390/ijerph182010832

**Published:** 2021-10-15

**Authors:** David Martín-Martínez, Pere Lavega-Burgués, Cristòfol Salas-Santandreu, Conxita Duran-Delgado, Queralt Prat, Sabrine Damian-Silva, Leonardo Machado, Pablo Aires-Araujo, Verónica Muñoz-Arroyave, Manuel Lapuente-Sagarra, Jorge Serna, Miguel Pic

**Affiliations:** 1 Motor Action Research Group (GIAM), INDEST, National Institute of Physical Education of Catalonia (INEFC), University of Lleida, 25192 Lleida, Spain; davidmartin.eurofitness@gmail.com (D.M.-M.); plavega@inefc.es (P.L.-B.); csalas@inefc.es (C.S.-S.); cduran@inefc.es (C.D.-D.); querian@hotmail.com (Q.P.); sabrinedamian@hotmail.com (S.D.-S.); leonardoed.fisica@hotmail.com (L.M.); pab_aires@yahoo.com.br (P.A.-A.); veronicarroyave15@yahoo.es (V.M.-A.); jserna@gencat.cat (J.S.); 2 Smart Performance & Sport Science, Faculty of Education and Sport, University of the Basque Country (UPV/EHU), 48940 Vitoria-Gasteiz, Spain; lapuente.manuel@gmail.com; 3 Development & Innovation on Conditioning & Exercise (DICFE) Research Group, National Institute of Physical Education of Catalonia (INEFC), University of Lleida (UdL), 2192 Lleida, Spain; 4 Motor Action Research Group (GIAM), South Ural State University Chelyabinsk, 454080 Chelyabinsk, Russia

**Keywords:** motor decisions-making, GPS, T-Patterns, acceleration, motor praxeology, role

## Abstract

The purpose of this study was to examine the players’ decisions-making in Marro (a Traditional Sporting Game) through a multimodal approach. Each player’s decision-making assumes specific accelerations and decelerations associated with different effort. The research objectives were: (i) to study the decision-making associated with the roles of Hunter and Hare; (ii) to know the physical effort by the roles (Hunters and Hares); (iii) to reveal T-Patterns in the multimodal strategic approach (integrated with decisions and different physical effort) with a direct incidence on the scoring by roles. The study was performed with 22 male and 2 female players aged 18 to 25 (M = 19.4; SD = 1.3). The Marro game was played by two groups for eight minutes. An observational methodology was used, through a type III design. The observational design was nomothetic, one-time, and multidimensional. An ‘ad hoc’ tool was built to ensure the data quality. Univariate analyses were performed using Crosstabs Command, with adjusted residuals (AR), Classification Trees (Chaid model) and T-Pattern Analysis (TPA). Significant differences were found between matches using the scoring (*p* < 0.001; ES = 0.26), role (*p* < 0.001; ES = 0.31), or the organic variables of the study, the speed (*p* < 0.001; ES = 0.73), the metabolic power and the acceleration/deceleration the speed (*p* = 0.023; ES = 0.43), while the predictive model pointed to the variable role (*p* < 0.001) as the main factor responsible for the model growth. TPA (*p* < 0.005) revealed differences attributable to internal logic in the yellow (first match) and orange (second match) teams, while organic variables were more changeable in the violet (first match) and green (second match) teams. This study advances the individualization of the decision-making process. These results may be useful to better understand the internal of functioning of the Marro game 360° since the use of various methodologies and variables (multimodal approach) provided original findings.

## 1. Introduction

Traditional sporting games (TSGs) constitute a unique family in the field of physical activity. TSGs are fundamental for the acquisition of basic life skills: cognitive, social, and emotional competences, and values and attitudes that define socially responsible citizens [1]. These are motor manifestations that result from cultural tradition, and therefore they often give rise to relationships and rules in accordance with habits and local customs. One of these TSGs is the Marro game (Prisoner’s Bar), practiced since the Middle Ages in Europe, in which two teams play against each other [1]. Players who leave their home can capture opponents who have gone out before them. However when a player is performing the role Hunter, trying to catch an opponent (Hare), he/she should notice that another opponent could have left home as a Hunter, in which case our protagonist would become his/her Hare. So, a player could be at the same time the Hunter of an opponent and Hare of another opponent. Once a player is captured, they are taken to the prison where they can be released by their teammates. The scoring depends on the number of captured players; so, there are two key actions in the game: capturing opposing players and saving teammates prisoners. To do this, it will be necessary to use individual and team motor strategies when leaving home and going through the roles of Hunter or Hare. TSGs, as Marro game, are based on a democratic agreement or, a social contract [1]. In order to play a TSG, all participants should respect the rights and prohibitions by the rules of the socio-motor game, which are organized in teams and which require players to engage in constant dialogue with others, whether they are members of the same team or rivals [2].

Any TSG has an identity card, an internal organization pattern, or internal logic, that is structured in relation to space, time, material and players, which allows its protagonists to continually adapt to a new motor situation [1,3].

The Marro game’s internal logic established by the rules requires the participants to perform motor interactions with the other protagonists according to the role in which they participate. At the same time, players are asked to use each of the spaces on the playing field in an intelligent way (e.g., to calculate the risky zones to be used when they became Hunters or Hares) in order to head to the opposite zone of the field to save prisoner teammates. Finally, it will also be necessary to conduct an intelligent relationship with time (each decision will have to be taken at the most appropriate moment, calculating the risk to be taken in each motor action, depending on the scoring, that is, the number of players captured by the player’s own team and the opposing team).

The Marro game becomes a laboratory of decisions, which in turn involve interpersonal relationships [1]. This is a game in which the motor conduct interpretation of opponents and colleagues, as well as the emission of messages to be decoded by other participants, are some examples of the processes that are activated. In this game, the adaptation to unforeseen events caused by the players’ information uncertainty confirms the high demand for the use of reflexive or cognitive skills [4].

The science of motor action or motor praxeology offers a theoretical framework to reveal the internal order that any game generates in aspects of as much interest as decision-making. Some key concepts are the universals or operational models that contain the internal logic of any game. For example, from the Scoring System model it is understood that the Marro is a zero-sum game, which generates complete but imperfect information according to game theory [4].

Through the universal corresponding to the network of changes in sociomotor roles, it is possible to identify the decisional burden of any TSG as the Marro. A role corresponds to the limitations, rights and prohibitions prescribed by the rules for one or several players [1]. In the Marro game there are three roles: Home (being in the protected area), field player (alive), and Prisoner.

Unlike team sports, in which attention is directed towards the decisions that are triggered around the ball (relationship with the material), in the Marro game, the players’s decisions are conditioned by an excellent management of timing. In this game, the “moment” of leaving home suggests the chance of having Marro (to be a Hunter) or receiving Marro (to be a Hare) over the opponents. In addition, participants may potentially become a Hunter (from a “x” rival Hare) and a Hare (from a “y” rival Hunter who has gone out later) at the same time. In these circumstances, players should choose the most suitable role in each sequence of play.

Reading (coding) and interpreting (decoding) the motor conducts of opponents and team mates confirm the importance of communication and processes involved, which at the same time are linked to extraordinary decision making. Reflective or cognitive abilities [5].

In the Marro game, decision making is associated with transitions of roles, which in turn lead to different relationships with team mates and opponents. Each of these roles triggers a set of minimal decision units (subroles). For this purpose, it may be convenient to incorporate the notion of a strategic role referring to a role integrating different groups of decisions. This is the case of the alive role, where any player could perform the strategic role of Hunter (and decides to pursue an opponent), Hare (when fleeing from the opponent), Neutral (when the decision on the opponents is not clear enough) and in Conflict (when some dispute and interruption occur between players). In this game, systematic observation allowed the identification of different subroles for each of the roles.

At the same time, these decisions and relationships trigger different energy commitments, associated with changes in space using accelerations and decelerations. The Marro game, like other collective games, is considered an acyclic or discontinuous game that requires the simultaneous participation of the aerobic and anaerobic systems to carry out with guarantees to the demands of the periods of sprint (maximum efforts) and moderate run (sub-maximum efforts). In the characteristic effort of this game, high intensity races are alternated with periods of rest or low intensity continuous races. This is the clue that it is to be considered a hybrid game, as far as conditional requirements are concerned [6].

Some adaptations on the organism could be related to the use of intervallic training with TSGs, as there are phases with very high intensities and periods of time where there are hardly any actions of medium to high intensity [7].

Normally, actions at maximum intensity are linked to changes in the score and, therefore, are determining actions when interpreting a relevant factor in the game as the final scoring.

Despite the advances in recent years, however it seems to be still scarce the line of research that addresses TSGs from the energy approach seems to still be scarce. According to [2], we will go further than previous investigations, to delve into different variables involved in the organic participation through Speed, Acceleration/Deceleration, and Metabolic Power *(MetPow)*. These variables belong to players’ external and Internal load, understanding external load as the parameters of the activity: Speed, Acc (acceleration), Dec (deceleration), and internal load as the physiological parameters of the player. The metabolic power contains, the internal load obtained indirectly, combining the external variables of speed and acceleration. For practical purposes, it cannot be considered as an internal load, despite the fact that the values obtained express the physiological parameters of the player.

The acceleration is the first variable corresponding to the mechanical intensity signals, which cause a change in speed over time, i.e., Acc (m/s^2^) = (V1 − V0)/(t1 − t0). It could also be understood as the amount of speed that changes in a given time. The characteristics of the concept of acceleration, are related to changes in speed that occur with respect to the time evolution. It would be relevant to know if the beginning of the action was from a static position, or from a specific speed. For example, since the absolute value of acceleration will be lower if the player is in movement than if the action begins at a lower speed or from a static position.

The player can obtain the same value of acceleration at two different initial and final speeds. However, the intensity generated would be very different, since at the same absolute value of acceleration it will tend to be lower when the initial speed is lower. The capacity of the player to change speed when moving is directly related to the capacity to manifest force in a specific way at high speeds, that is, it depends on coordinative and conditional aspects.

Acceleration, as a study variable, also provides the actions with the negative value: decelerations or braking of the player. When the decelerations are of maximum intensity, there is a direct relationship between the initial speed with which the action starts.

According to [8], the maximum Acc. is achieved in the first second and, on the other hand, the maximum deceleration is achieved between 0.5″ and 1″. The intensity is defined not only by the value of the Acc, but also by the time it takes to reach it, since if the player manages to make the deceleration in a shorter period of time, it means that he/she manages to apply that same force, but in a faster way, and therefore the player achieves higher power values, and a higher intensity. It also seems that players usually reach a speed of 3–4 m/s at maximum accelerations, although it can reach up to 5 m/s in those players who probably have more capacity for speed. The maximum deceleration is usually reached between 2 and 5 m/s, obtaining higher values with those players who show greater values of explosive force in eccentric contractions.

*Metabolic power* (MetPow) is another’s of the variables in our study. MetPow *is* an intensity signal [9] derived from the player’s instantaneous speed [10] and acceleration [11], which indirectly determines the amount of adenosine triphosphate (ATP) required by the activity being performed.

If the speed of the action is slow and constant, the metabolic power values are low. On the other hand, when the changes in speed is higher both the values of the Acc and the MetPow are increased too. When the player slows down, instead of being 0, the MetPow values obtained are lower due to the low energy requirement [9].

Through this MetPow intensity signal, it is also possible to calculate the oxygen consumption (VO2) in an indirect way. This data allows to associate it with a concept of energy expenditure and to analyse the values obtained in order to interpret the game and to know which energy aspects it develops at an aerobic or anaerobic level.

These values allow a progressive evolution of the load for the physiological demands of this game, based on the intermittence of the player’s effort. These actions are classified in the three categories with MetPow values above 20 W-Kg^−1^. The training schedule determines that less than 5% of the actions performed by the sample of players in eight minutes are of high intensity. However, on the other hand, a relationship is established between these actions and the score variable that determines the final result of the game. With the data obtained, a training of the players can be proposed by simulating the energetic manifestations of the relevant situations of the game as Campos and Lapuente’s proposed (2018) (adapted from [7], the short interval training (<45″): Sprint Interval Training (SIT), and Repeated Sprint Training (RST).

It is possible to define individual intermittence profiles, in order to individualize this training according to the physiological demands obtained. These demands will be variable, depending on various factors such as the scoring, rival attitude, teammate attitude, etc. However, if similar conducts are established in different games, individualized training sessions can be created for each player according to his intermittence profiles. These individualized training tasks are obtained from the relationship that exists between different actions where the player exceeds a threshold of high energy expenditure and the pause that exists between both actions. This information allowed us to program the type of training that the player has to experience, to establish a direct relationship with the strategic needs during the game by each player.

Physical effort, decisions and motor interactions are different dimensions of the same multimodal or polyhedral phenomenon: the motor conduct promotes a multidimensional 360° approach: through an organic, decisional and relational nature [2].

From this theoretical framework, the objectives of the present study were performed according to two matches (four teams): (a) to study the decision-making associated with the roles of Hunter and Hare; (b) to know the physical effort in the motor actions of the roles of Hunters and Hares; and (c) to reveal T-Patterns in the multimodal strategic chains (integrated with decisions and different physical effort) in the motor actions of the Hunter and Hare with a direct incidence on the scoring.

## 2. Materials and Methods

The design was based on an Observational Methodology [12,13] already contrasted by studies in sports and TSGs [2]; located in its quadrant III [14,15]. The selection of this quadrant was justified by the following reasons: (i) the study of the decisional collectivity (teams) when playing suggests a nomothetic character; (ii) the inexistence of a monitoring plan (just although a specific recording was made); and (iii) the use of different criteria (roles) and categories (subroles) reminds that it is a multidimensional recording system [15,16].

### 2.1. Participants

This study involved 24 players (girls = 2, 8.3%; boys = 22, 91.6%), undergraduate students in the first year of physical activity and sports sciences at the University of Barcelona, Spain, who were enrolled in the pedagogy course. To practice the Marro game (two matches) players were randomized in order to form teams. The participants’ decisional and energetic intervention was studied for eight minutes (480 seconds) during the game.

### 2.2. Variables

In this research six categorical variables were examined: scoring, strategic roles, subroles and speed, acceleration/deceleration and metabolic power were used.

The ***Score*** variable used the following coding: tie (ZE), +1 (ON), +2 (TW), +3 (TH), +4 (FO), +5 (FI), +6 (SI), +7 (SE), +8 (EI), +9 (NU), +10 (TE), −1(NO), −2(NT), −3(OH), −4(NFO), −5(NFI), −6 (NS), −7(OE), −8 (NE), −9 (NU), −10 (NT).

The strategic role variable was coded into five categories:

(a) *Home* (HM), a player who is at home area. In this specific area, players can neither capture nor be caught.
(b) *Prisoner* (PR), a player who has been caught while they were out of home. Prisoners players were located on one of the sides (1.5 m from the home area of the opposing team).

For the living role (being away from home), systematic observation made it possible to identify four strategic roles associated with different decision-making by the living player:

(c) *Hunter* (HN), a player who chases an opponent.
(d) *Hare* (HR), a player who runs away to avoid been captured.
(e) *Neutral* (N), a player who is in a neutral situation, without directing his decision towards capturing or running away.
(f) Conflict (CF), a player in a dispute against an opponent, being interrupted inhis/her participation in the Marro game.

The variable ***Subrole*** corresponds to the minimum decision units.

When the decision-making does not change the score by roles, it was decided to identify them with the name NI (no impact on the score). In this way, the following categories were coded:

Home Role (HM):

(a) Subrole NI: no incidence on score;

Hunter (HN):

(a) ZNI: no impact on score;
(b) ZC Hunter that is at the moment of capturing.
(c) ZP is caught: Hunter who is chasing (running).
(d) ZS Saving: a Hunter who touches the hand of one of the prisoners who is joined in a chain.

Hare Role (HR):

(a) LNI: no incidence on score;
(b) LC Capture: Hunter who catches a rival player;
(c) LP She is caught: Hare that when fleeing is caught by a rival Hunter;
(d) LS saving: Hunter who is currently saving his/her fellow Prisoners.

Neutral (N):

(a) No impact on score (NNI)

Prisoner Role (PR):

(a) NIP: no impact on scoring;

Conflict Role (CF):

(a) FF Conflict: Players who, for whatever reason, do not play. The stoppage of the game can be partial (players who are in conflict, but the rest are still playing) or total (the entire game is paralyzed). Whether it is total or partial, it will be categorized “in conflict” until the moment the game is restarted with the motor decision-making that is taking place at that moment of restarting the game.

By using devices with a global positioning system (GPS), three variables were recorded referring to the biological or energetic dimension of the players: Speed, Acceleration/Deceleration, and Metabolic Power (MetPow).

The variable Speed (m/s) originated six categories of thresholds (Table 1). The values were differentiated for both genders according to [17].

The *Acceleration* variable corresponded to the mechanical intensity signals that caused a change in speed over time, i.e., Acc (m/s^2^) = (V1 − V0)/(t1 − t0). Table 2 shows the thresholds observed and codified for this variable.

The metabolic power (MetPow) was another variable related to an intensity signal derived from the player’s instantaneous speed and acceleration. MetPow indirectly determines the amount of ATP required by the activity being performed. The thresholds corresponding to this variable are identified in Table 3.

### 2.3. Procedures and Instruments

#### 2.3.1. Application of the Marro Game

A protocol was applied to homogenize the application of the procedure in practice. The explanation of the game was written and presented to the teacher for the groups before carrying out the experience in order to neutralize the influence of the explanation of the game.

Although Marro can be played in different ways, in this study, it was performed with the following rules: two teams with an equal number of players were placed in a protected area (home) behind a line at one end of a rectangular field. Each player who left the house could chase and capture (as a Hunter) the opponents (as Hares) who had left before him/her. However, if an opponent left his/her house after him/her, the latter had “Marro” on him/her and would become his/her Hunter, so he/she would become his/her Hare. When a player caught a Hare, he/she would take he/she to the prisoner area, in a side at 1.5 m where he/she would be placed forming a chain (holding hands) with the rest of the prisoners of his/her team. If a fellow Hunter or Hare managed to touch a prisoner on the chain, he saved all the prisoners, although they could be recaptured by any opponent, before returning home. The team that had captured all the opponents before, or that had captured a larger number of prisoners after eight minutes, won [2].

Before starting the match, participants performed a series of exercises to adapt to the intensity of the physical effort of the match. An 8-min Marro game was then played and recorded with two cameras for observational analysis.

On the other hand, during three sessions prior to the final recording, simulated recording cameras were placed to reduce the reactive effect produced in individuals exposed to filming cameras. The students experienced the game for the first time during the session prior to the day of the recording, and ten minutes were made available so that doubts related to strategic interpretation or rules of the game could be dispelled.

#### 2.3.2. Use of Cameras, GPS Devices, and Specific Software

After explaining the game rules, a GPS device was handed out to each player having identified the device ID and the student ID.

GoPro cameras and STAT Sports GPS devices, model Apex Pro, were employed; while the Excel tool, the data analysis programs Spss v.25 and Theme v.6 [18,19] were used.

Once the data were collected by the location systems, the StatSports software was employed to download the records. When the data were exported, the different drills, or time interval cuts from the different parts of the practice, were performed. The first cut made was from the time the device was switched on until the starting of the warm-up. All participants performed a pre-practice activation, which was logged and directed by those responsible for the study. The second record was obtained by recording five vertical jumps by each of the participants, to avoid any errors, and to make it easy to be observed the beginning of the Marro game. Finally, the end of the recording was made when the game ended, after eight minutes all players performed five vertical jumps.

Once we had the different cuts of the data export, we proceed to analyse the drill of the game, and each of the data obtained in those eight minutes.

#### 2.3.3. Creation of a Database Obtained from GPS

Recording events were placed on an excel sheet. To build up this database, the first column corresponded to ‘time’, each row being reserved for the second scale. Therefore, 480 rows were used per player (8 min). While no changes were made to the original format of the database in order to apply the statistical strategy of the decision trees, nevertheless, all organic variables were transformed into a category format before the crosstab command were set up. In order to perform TPA, the events occurrences repeated in more than one consecutive occasion were deleted [2]. Thus, if during the first minute, the categories (events) appeared (for example, A, A, B, B, C, C) it would mean that the player performed the category A for 3 s, the category B for 2 s and category C for 3 s. For this reason, given that the concrete second the category (occurrence) began. It would be possible to calculate its specific time (Interval duration) since the end of a category coincided with the beginning of the next one. In this way, the repetitions of categories were deleted from raw data following two unavoidable premises (Table 4): (a) the first time that each event appeared was considered original and, therefore, included in transformed data, and on the other hand, (b) Keeping the original temporal reference [20]. This transformation allowed the use of TPA as well as a significant database reduction. This type IV data is particularly relevant when it comes to knowing the T-data structure [21], already applied in the TSGs scenario [22]. This procedure was applied with Python’s programming tool [2].

#### 2.3.4. Observation System Validation by Roles, Subroles, and Data Quality

Different strategies were followed to address the data quality in events (occurrences). Firstly, the mixed ‘ad hoc’ system was built with the expertise supported by the GIAM research group. All the categories and criteria were defined (concept and his opposite), then put into practice by identifying them in the recordings. The game events were done directly on an excel sheet (Microsoft, 2010). Finally, two expert observers were selected to carry out a focal follow-up of each player, with a duration of 8 min. Highly reliable statistical correlation coefficients were achieved. The inter and intra Cohen’s kappa coefficient (κ) were found to be higher than 0.80, thus ensuring the data quality of the events. On the other hand, to verify the quality of the GPS devices, instantaneous signal quality indicators were used, with a number of satellites above nineteen, among other indicators. It is also important to assess the facilities where the signal was obtained, in case they have metallic structures, high buildings, stadiums, etc. and this study was carried out in an outdoor sports facility without buildings or stands around.

### 2.4. Data Analysis

#### 2.4.1. Use of Crosstabulations

In the present study, crosstab command (*p <* 0.05) by pairs of variables were applied, taking into adjusted residuals (ARs) > 1.96 or <−1.96 [23] as well as effect sizes through Cramer’s V test. According to [24], to interpret the value of the effect size it was followed: 0.10 = small effect, 0.30 = medium effect, and 0.50 = large effect.

#### 2.4.2. Decision Tree

A supervised learning model or decision tree was applied, (Chaid model) [25]. Starting from the initial variable (dependent variable), the rest of the variables were considered predictive variables of the model; next requirements were followed: (i) cross validation, (ii) Pearson’s chi-squared was used, (iii) the maximum levels of the model was 5, (iv) the number of cases grouped in parent and child nodes fluctuated between 100 and 200 cases. All the analyses were carried out with the SPSS v.25 tool (SPSS Inc., Chicago, IL, USA).

#### 2.4.3. T-Pattern Analysis

T-Pattern Analysis (TPA) [22,26,27,28] was used to reveal structural regularities, invisible to a superficial eye. TPA is a novel algorithm to find out both sequential and temporal regularities. TPA is a multivariate technique based on the lengths interval (time) and sequences of events (occurrences). Following [26] ‘if A is an earlier and B a later component of the same recurring T-pattern, then, after an occurrence of A at t, there is an interval [t + d1, t + d2] (d2 ≥ d1 ≥ d0) that tends to contain at least one occurrence of B more often than would be expected by chance’. The search parameters when applying TPA were: (i) Level of significance for the critical interval (*p <* 0.005) [29,30], (ii) The most complex T-Patterns were based on 5 occurrences; (iii) Selection of free heuristic critical interval setting [31], (iv) TPA detection were validated by simulation, through data randomization [29,32].

#### 2.4.4. Frequency Areas

Finally, in order to construct the frequency areas the accumulations of a minimum of 35 frequencies between both team were selected, in their respective matches, with differences between team greater than (±4.0 adjusted residuals).

## 3. Results

Descriptive tables (Crosstabs) allowed us by pairs the associative significance (*p* < 0.05) and effect size. On the other hand, the classification tree showed the strength of the variables to predict the similarities or differences by matches and teams. The use of TPA (*p* < 0.005) identified to 360° multimodal strategic chains (score, role, subrole and physical values) of both Marro matches, according to different scores and roles. Finally, the frequency areas showed the differences between the matches and the teams from 360° multimodal approach. Below, the results of each of these sections were described.

### 3.1. The Scoring Variable in Both Matches

Two Marro’s matches were taking into account for analysed (11,100 s) through the 4 different teams’ scoring: (Orange vs. Green, OG; Yellow vs. Violet, YV). Both matches were divided into 5760 s in the NV match and 5340 s in the AL match. This second match (AL) ended before the 8 min due to one of the teams achieved the goal of capturing all their opponents.

**Figure 1:**
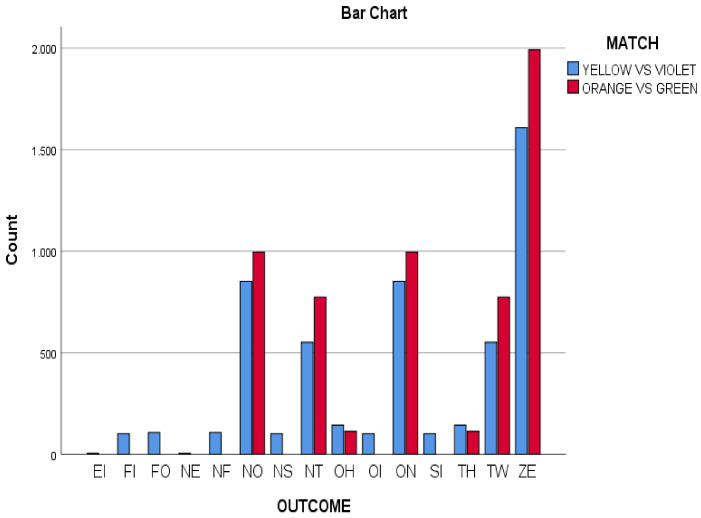
Outcome variable in both matches (Yellow vs. Violet, Orange vs. Green).

Significant differences were found (*p* < 0.001; Cramer’s V, Effect Size (ES), =0.26) when comparing the scores of the two matches OG and YV (see Figure 1). In both matches, the most frequent result was ZE (O, tie) *n* = 3600; 67.41% (YV match: *n* = 1608; 30.11% and OG match: *n* = 1992; 34.58%). The second most found score in both matches were ON (+1) and NO (−1). Both scores were found more times in the match OG, ON (1) 996 and NO (−1) 996; (34.58%). The third most important results (*n* = 1326) were TW (2) and NT (−2) (YV: *n* = 1104; 20.67%; and OG: *n* = 1548; 26.87%). The OG match always had a greater presence of these five types of scores (ZE, ON, NO, TW, NT), with respect to the YV match. Finally, it was noted that some scores (EI, FI, FO, etc.) were only observed in YV match.

In order to characterize the teams, situations with the greatest margin of difference in the score were investigated. The emergence of the most complex T-patterns (dendrograms) emerged in the first match with TW (score 2) and not (−1), while in the second match the TW score was found again (2), but also on (1) and NT (−2).

### 3.2. Roles Used by Both Matches

**Figure 2:**
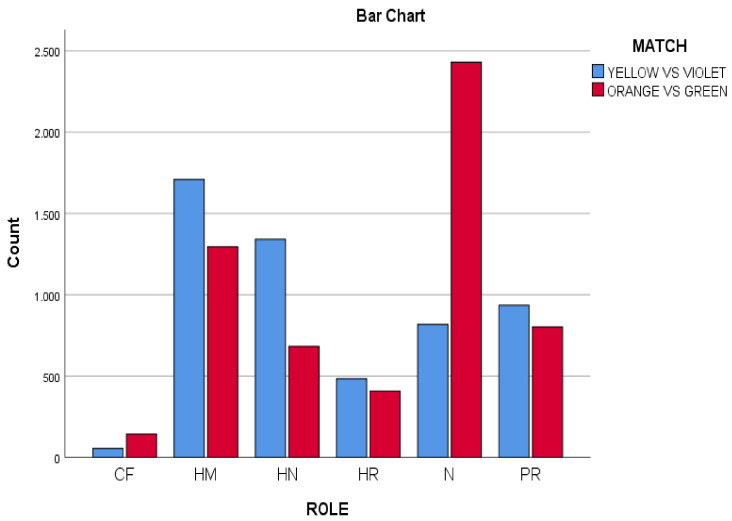
Role variable distributed by scoring in both matches (Yellow vs. Violet, Orange vs. Green).

Significant differences were observed (*p* < 0.001; ES = 0.31) when comparing the scores of the two matches OG and YV (see Figure 2). The HM (home) role was the most used in the YV match *n* = 1709; 32%), while the N (neutral) role was the most used in the OG match (*n* = 2430; 42.18%). It should notice that HM and N roles are passive roles, although the type of decisions-making are potentially unequal in the N and HM roles. In the N role, the player was out of home, and at any moment he or she can go on the run or chase. In contrast, in the HM role, the player was protected at home and cannot make either decision.

YV and OG were also different in the second role. In YV the players were more active in decision- making, since a superiority of the role HN (Hunter) was found (*n* = 1341; 25.11%), with respect to the roles PR (prisoner) (*n* = 933; 17.47%) and N (neutral) (*n* = 817; 15.29%).

In both matches, the HN (Hunter) role was more present than the HR (Hare) role. Despite sharing that regularity, the HN role was more prominent in the YV match than in OG (Hunter role: YV: *n* = 1341; 25.11% vs. OG: *n* = 682; 11.84%). These differences between both matches were less in the HR role (Hare YV role: *n* = 483; 9.04% vs. OG: *n* = 408; 7.08%).

In both matches, the role of PR (prisoner) was similar (Prisoner role: YV: *n* = 935; 8.42% vs. OG: *n* = 802, 7.22%).

Another finding of the study neither of the two matches were conflicting. The role of CF (conflict) was scarce (YV: *n* = 55; 1.02%, vs. OG *n* = 143; 2.48%).

Regarding subroles, it was observed how the decisions that did not directly affect the score (NI = no changes were detected on the score) were the most prominent

### 3.3. Subroles Performed in Both Matches

**Figure 3:**
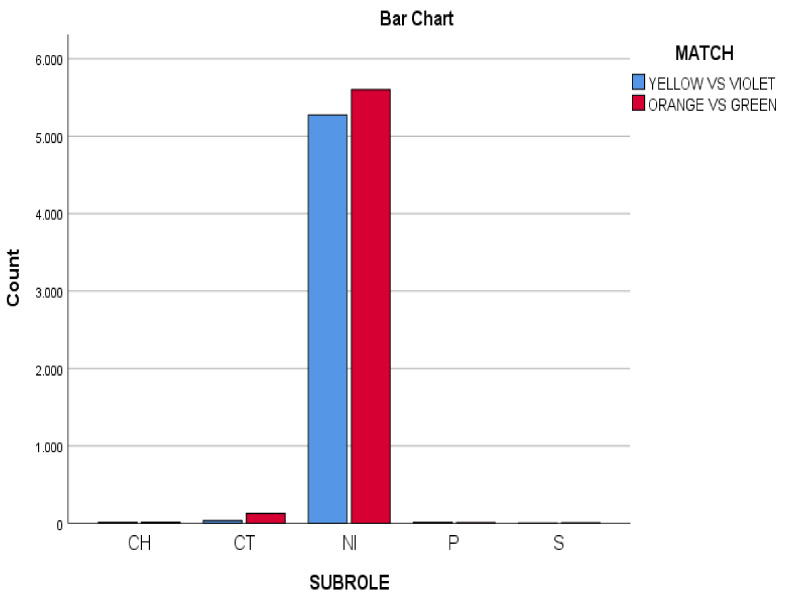
Subrole variable in both matches (Yellow vs. Violet, Orange vs. Green).

Concerning the subroles (see Figure 3), the decisions-making without direct incidence (changes) on the scoring (NI) were the most frequents (*n* = 10,874; 97.96%). This regularity was similar in both matches (YV: *n* = 5274; 98.76%, vs. OG *n* = 5600; 97.22%). The subroles CH (capture) (*n* = 28; 0.25%) and S (save) (*n* = 10; 0.09%) decisive for scoring only occurred on 38 occasions. This scarce presence was also observed in each match CH (YV: *n* = 13; 0.24%, vs. OG *n* = 15; 0.26 %) and S (YV: *n* = 13; 0.05%, vs. OG *n* = 15; 0.12%).

### 3.4. GPS Devices in Both Matches

**Figure 4:**
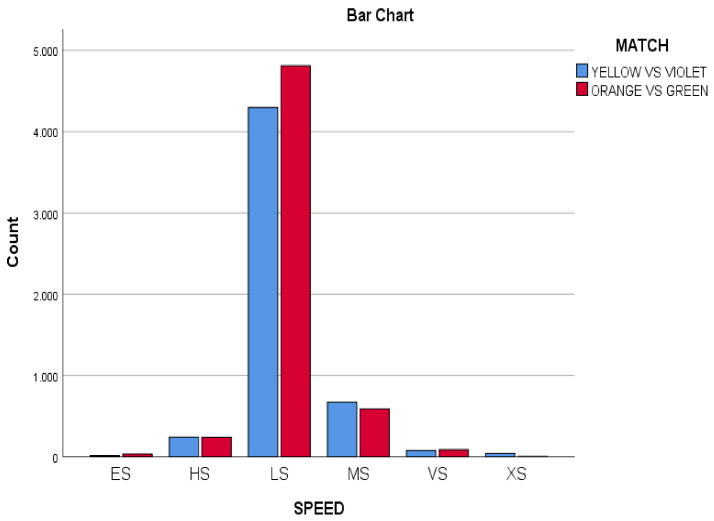
Speed in both matches (Yellow vs. Violet, Orange vs. Green).

**Figure 5:**
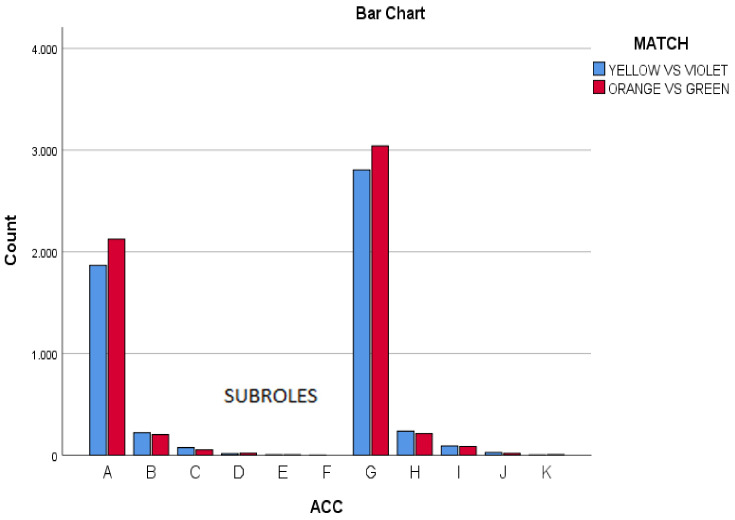
Acc in both matches (Yellow vs. Violet, Orange vs. Green).

**Figure 6:**
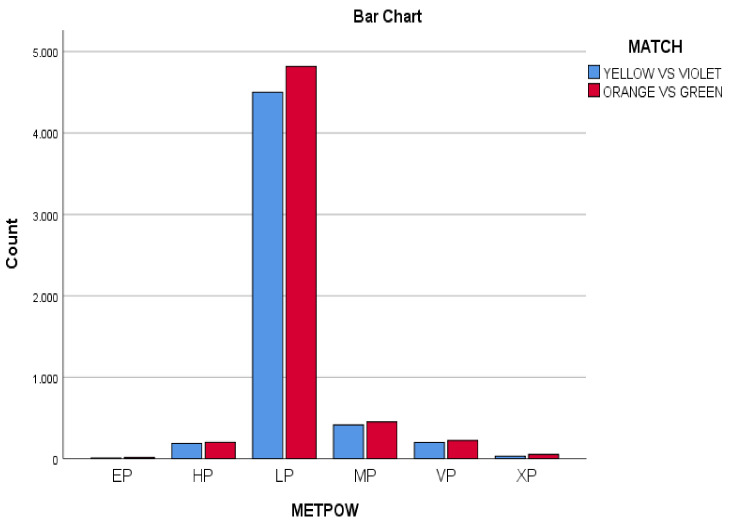
MetPow in both matches (Yellow vs. Violet, Orange vs. Green).

It was observed a great predominance of the less intense values (>80%) in the three variables: Speed (Figure 4), Acceleration/Deceleration (Figure 5) and Metabolic Power (Figure 6), in both matches (YV and OG).

The minimum values of the three variables were higher than 80% of the total sample in each of the variables. Speed actions, there are less than 7 km/h per second (6 km/h in the case of girls), (*n* = 4297; 80.46%) in the YV match and (*n* = 4811; 83.52%) in the OG match. In both matches, the MS (medium speed, between 7 and 13 km/h) and HS (high speed, between 13 and 18 km/h) actions had a similar protagonism (YV match: MS (medium speed) *n* = 670; 12.54%; OG match: *n* = 587; 10.19%). The three maximum speed categories (VS, XS and ES), in the two matches represent less than 2.5% of the total actions, (YV match: *n* = 131; 2.27%; OG match: *n* = 123; 1.10%).

Regarding the speed variable, it was observed that most of the match actions (>80%) were performed at low speed (LS = men < 6 km/h; women < 7 km/h) in both matches (LS: YV: *n* = 4297; 80.46%, vs. LS OG *n* = 4811; 83.52%). The average speed values (MS = 7 to 13 km/h) corresponded to 10% of the playing time of both matches (MS: YV: *n* = 670; 12.54%, vs. MS OG *n* = 587; 10.19%). Finally, high speed actions (HS from 13 to 18 km/h) were less than 3% of the actions of both matches (HS *n* = 131; 2.27%, vs. MS *n* = 123 times; 1.10%).

In the section on accelerations and decelerations, it was described how the same manifestations of the players were observed, despite they were playing in two different contexts, with different decisions-making and motor actions. In the two matches studied, the actions with less speed (−1 m/s to 1 m/s) occupy 88.61% of the actions with 9836 times of the total (*n* = 11,100). Actions with acceleration range 3 to 5 km/h and those with deceleration range −3 to −5 km/h represent 0.83% of the total actions analysed (*n* = 93), indicating that actions with maximum intensity of this variable do not often appear.

It was also observed that in both matches the values of accelerations and decelerations had a similar protagonism. Most actions were of lower speed (AC/DC Low −1 m/s to 1 m/s: *n* = 9836/11,100; 88.61%). Acceleration actions from 3 to 5 km/h, and deceleration actions from −3 to −5 km/h were very scarce (*n* = 93; 0.83%).

The relationship between the values of maximum acceleration and maximum speed at that particular moment, obtained from the players in the study, describes a linear relationship that allows us to identify a linear slope, to find out, the acceleration capacity with respect to their speed. With this data, not only the performance of the players could be evaluated, but also it could be prescribed training to each subject.

Relationships were established in the study of the values of metabolic power obtained in the players by two matches. Where the average difference of the variables studied was 0.20, it is then determined that the same exact values were reproduced, despite studying two different situations of the same game. The LP category (low power, from 0 to 10 w/kg) appears in more than 83.95% of the total, being (4501) times; 84.28% in the YV match, and (4818) times; 83.64% in the OG match. In the comparison with each of the variables, there was a direct relationship between the two matches, where the greatest difference between the values of MetPow (metabolic power) was 0.66 in the variable that appears most in LP (low power). The values in the YV heading were LP (*n*= 4501; 84.2%), MP (medium power) (*n* = 415; 7.77%), HP (high power) (*n* = 187; 3.5%), VP (very high power) (*n* = 200; 3.74%), XP (maximum power) (*n* = 30; 0.56%), and EP (extreme power) (*n* = 7; 0.13%), unlike the OG match where the values of LP (*n* = 4818; 83.6%), MP (*n* = 451; 7.82%), HP (*n* = 202; 3.50%), VP (*n* = 223; 3.87%), XP (*n* = 51; 0.88%), and EP (*n* = 15; 0.26%).

The two matches yielded the same values for the different categories: LP (low power), MP (medium power), HP (high power), YP (very high power): YV match (*n* = 4501; 84.2%); OG match (*n* = 4818; 83.6%).

### 3.5. Predictive Capacity Variables by Teams

Multimodal results 1. Predictive capacity variables on the multimodal conduct in the two matches.

**Figure 7:**
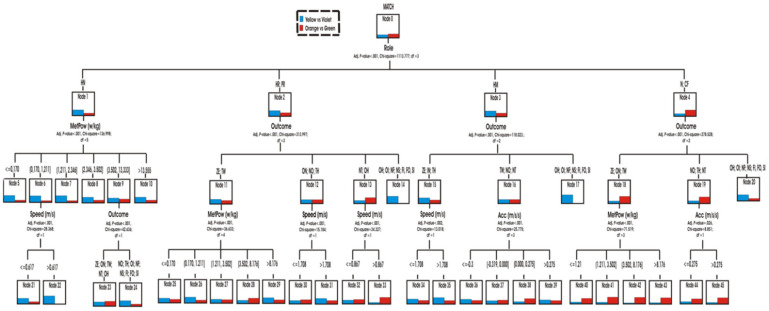
Predictive capacity of the scoring variable (two matches). Note. For easier reading of this figure, this classification tree has been segmented into different images, which are shown below.

Through the hierarchical segmentation technique or classification tree, the predictive capacity of the score variables (role, speed, accelerations/decelerations and metabolic power) was explored to reveal the similarities or differences in the multimodal behaviour of both matches (Figure 7).

**Figure 8:**
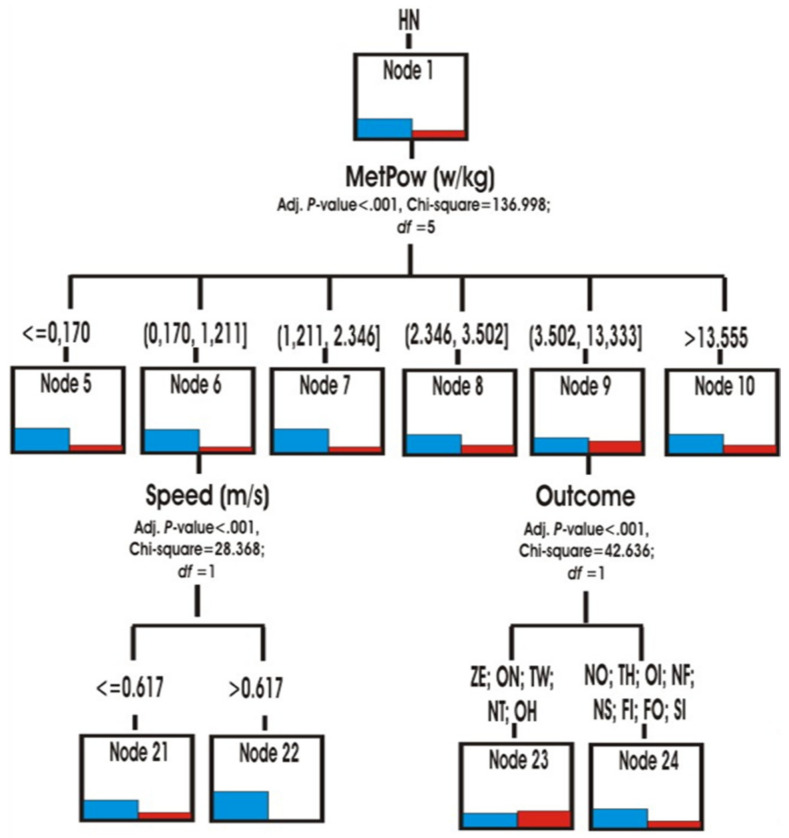
Predictive capability of the Hunter role.

**Figure 9:**
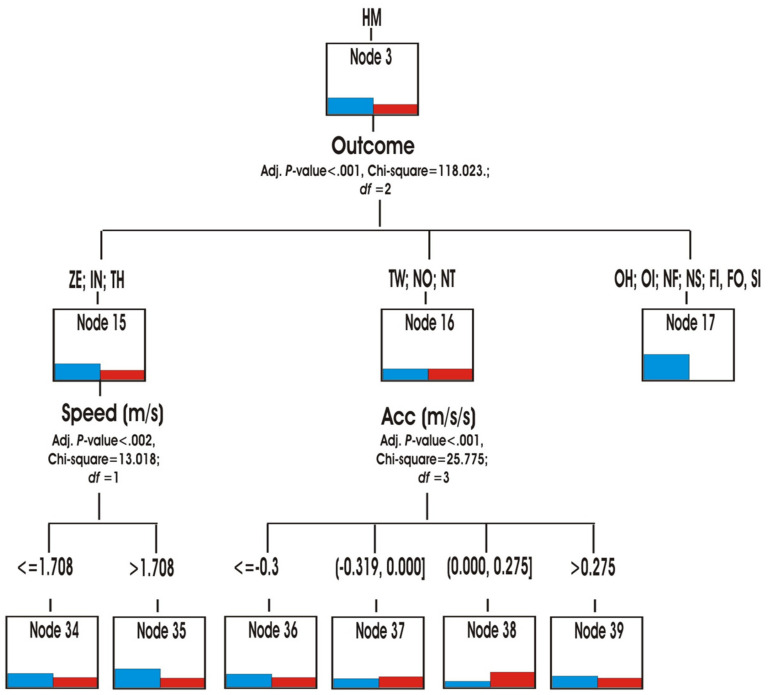
Predictive capability at Home Role.

**Figure 10:**
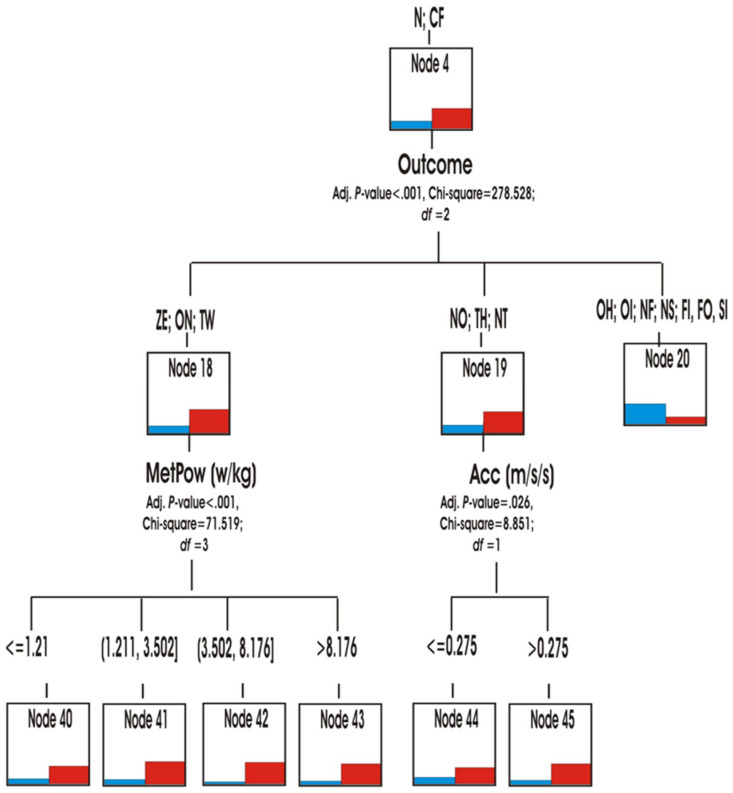
Hare and Prisioner Role Predictability.

The role was the first predictive variable (*p* < 0.001; chi-square 1,113,777, df = 3) in both matches. The teams in YV match, spent more time in the Hunter role HN (node 1) and being at home HM (node 3) (Figure 8 and Figure 9) than the teams in OG match. They also spent slightly more time in the roles of Hare HR and Prisoner PR (Node 2). In contrast, the teams in the OG match participated more in the Neutral N and Conflict CF roles (node 4) (Figure 10).

The second most predictive variable when comparing both matches was the score. Score was the explanatory variable in three of the previous nodes.

The physical variables were the least present in the tree as predictive variables: metabolic power (three times, once at the second level), speed (three times at the third level) and acceleration/deceleration (twice at the third level).

The main predictor variable for the roles Hare HR and Prisoner PR (Node 2) was the score (*p* < 0.001; chi-square 313.997, df = 3). It was observed that the teams in the AL match participated for longer than those in the OG match with the Z and TW tie scores (Node 11). In this case, the next predictive variable of both matches was the metabolic power (nodes from 25 to 29).

In parallel, the YV match participated much more than OG in actions with the scores OI, NF, NS, NE, FI, FO, SI (node 14). The OG match played longer than YV with the NT and OH scores (node 13). The two matches had a similar prominence when the score were ON, NO, TH (node 12). The regularities found in the nodes 12 and 13 were specified with more detail when the following speed prediction variable was used.

When the teams were in the HM home role, the tree identified the score variable that originated three types of behavior (nodes 15, 16 and 17) according to different scores (*p* < 0.001; chi-square 118,023, df = 2). The teams of the YV match spent more time than those of the OG match in the role of HM with the scores of ZE, ON, TH (node 15). In this case, the next predictor variable was the speed to go from one place to another in the home or to go out. It was observed that, in the YV heading, the actions were done with greater speed (V > 1708 m/s) than in the OG heading (*p* = 0.002; chi-square 13,018, df = 1). The teams participated in different accelerations/decelerations when they intervened with TW, NO, NT (node 16) scores (*p* < 0.001; chi-square 25,755, df = 3).

**Figure 11:**
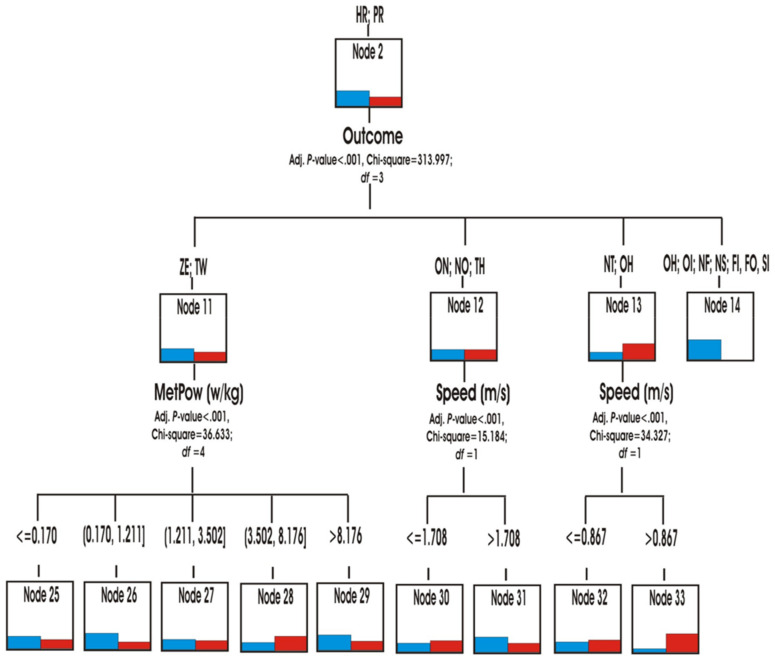
Role Neutral and Conflict Predictability.

It was also observed that the teams of the OG side spent more time than YV adopting the neutral N or conflicting CF roles (node 4) (*p* < 0.001; chi-square 278,528, df = 3). This superiority was also shown in two types of scoring: ZE, ON, TW (node 18) and NO, TH, NT (node 19). In contrast, the trend was reversed with the other scores OH, OI, NF, NS, FI, FO, SI (node 20). In order to predict the transition to other roles, the tree predicted the metabolic power (node 18) or the acceleration/deceleration (node 19) variables as predictive in both matches (Figure 11).

Finally, the main predictive variable for the Hunter role HN was the metabolic power (*p* < 0.001; chi-square 136.998, df = 5). The match YV showed recorded higher MetPow values in most occasions with respect to what happened in the OG match. This superiority was mainly identified in energy actions with values between 0.1 and 1.21 w/kg (node 6). For this node, speed also intervened as a predictive variable. It was observed that the speed of the actions was higher in the match YV with respect to the OG (*p* < 0.001; chi-square 26.368, df = 1). In the remaining cases, the two matches intervened with a similar MetPow in motor actions with values ranging from 3.50 to 13.5 w/kg (node 9).

Multimodal results 2. T-Patterns through the strategic multimodal by teams in the Marro game.

**Figure 12:**
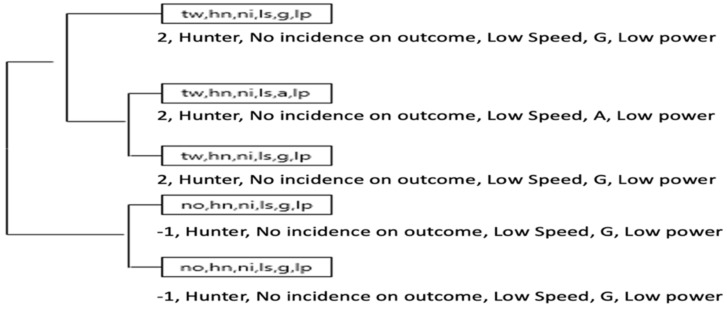
The most complex T-Patterns detected by Yellow team.

**Figure 13:**
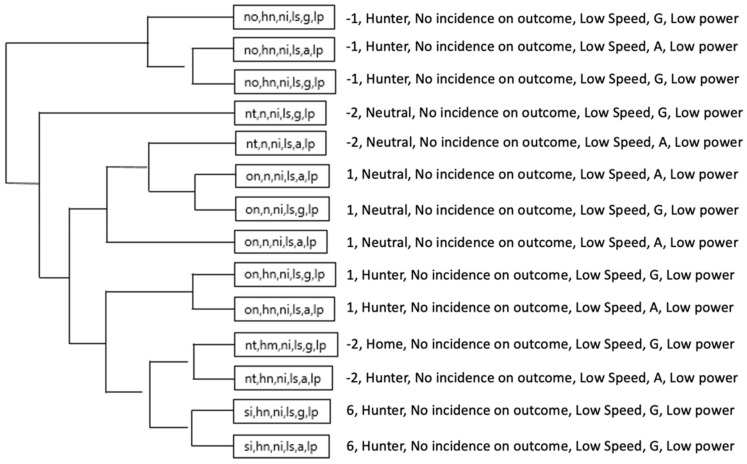
The most complex T-Patterns detected by Violet team.

Figure 12 and Figure 13 shows the strategic multimodal chains in both matches. In match YV with the TW scoring (+2), the variable chains (frequencies) most used by the Yellow team are (tw, hn, ni, ls, g, lp), therefore, with TW score (+2) and HN role (Hunter) and subrol NI no incidence on the score, with minimum values of speed, acceleration and metabolic power.

The strategic chain (no, hn, ni, ls, g, lp) by the Violet team, in which the Hunter role appears with a NO scoring (−2), and with the minimum values of speed, acceleration and metabolic power. On the Violet team, the ON (1) score also appear, linked to a N (neutral) role (Hunter).

Description of the T-Pattern analysis:

In the Yellow team, 2 strings were identified with result in favor of 2 (result +2, Hunter role, subrole no incidence score, low speed, Acc/Dec 0–1 m/s, MetPow 0–1 w/kg), after which followed the sequence of result in disadvantage of 1 (result −1, Hunter role, subrole no incidence on outcome (scoring), low speed, Acc/Dec 0–1 m/s, MetPow 0–1 w/kg).

Violet Team:

In general, there was some regularity to be used the Neutral role through the following outcomes (scoring) −1, −2 and +1, while the Hunter role in favor +1 outcome (scoring). Finally, there was a tendency for the home role to be used if the result was at a disadvantage of 2.

**Figure 14:**
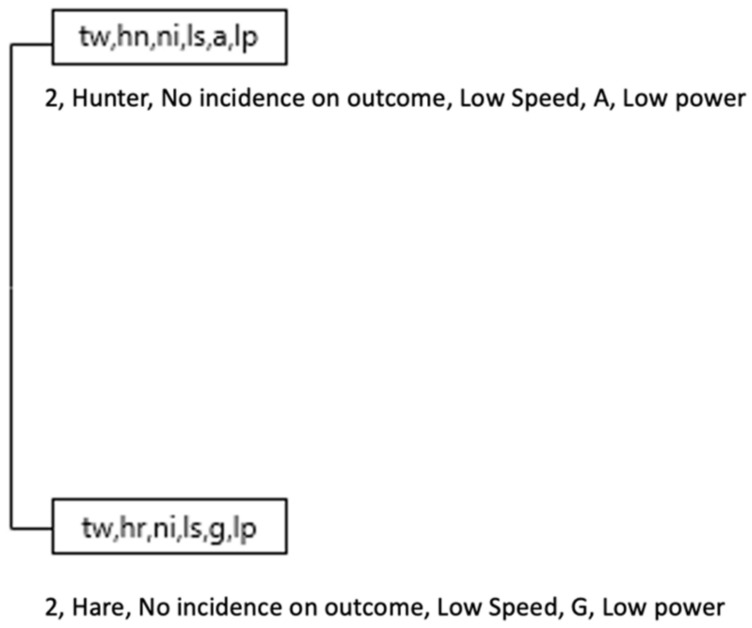
The most complex T-Patterns detected in Yellow team by Hare roles.

**Figure 15:**
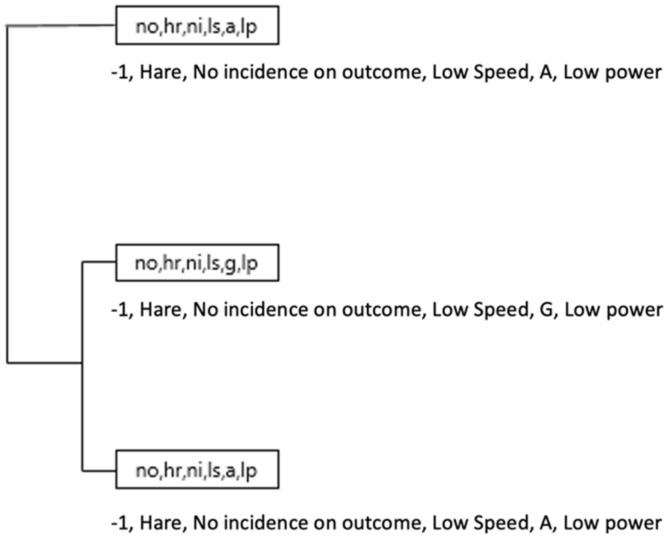
The most complex T-Patterns detected in Violet team by Hare roles.

Score:

On the other hand, while in the Yellow team, with result TW (+2) the Hunter role, also the HR (Hare) role appeared (Figure 14 and Figure 15).

In the Yellow team 2 chains were identified with result in favor of 2 (result +2, Hunter role, no incidence subrole, score, low speed, Acc/Dec 0–1 m/s, MetPow 0–1 w/kg), after which followed the sequence of the same result with the appearance of the role Hare (+2, Hunter, no incidence outcome, low speed, Acc/Dec 0–1 m/s, MetPow 0–1 w/kg and (+2, Hare, no incidence outcome, low speed, Acc/Dec 0–1 m/s, MetPow 0–1 w/kg).

The Violet team offered its own particularities with a regularity in the Hare role with the marker at −1 and the subrole of no incidence outcome, accompanied by physical effort variables of low speed, Acc/Dec 0–1 m/s, MetPow 0–1 w/kg.

In the OG, it was mainly observed that the strategic chains of the Orange team (on, hr, ni, ls, a, lp) and (on, hr, ni, ls, g, lp), associated an ON score (+1) and HR Role (Hare) as well as minimum values of speed, acceleration and metabolic power. Then, the same score appeared in Role HM (home) and HR (Hare) unlike the Violet team with strategic chains that prioritized a NT score (−2) associated to Role N (neutral) and Subrole NI (no incidence in the score).

**Figure 16:**
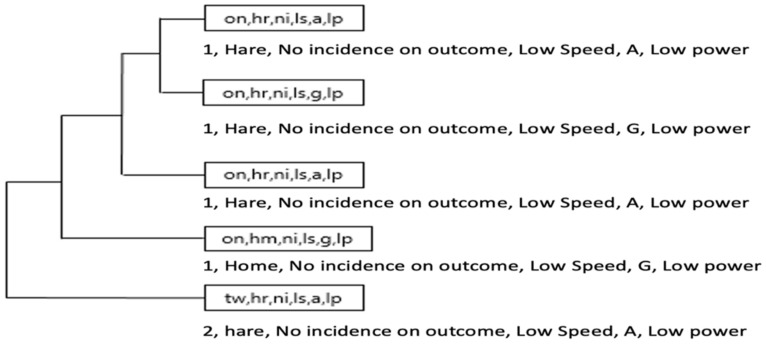
The most complex T-Patterns detected in Orange team.

**Figure 17:**
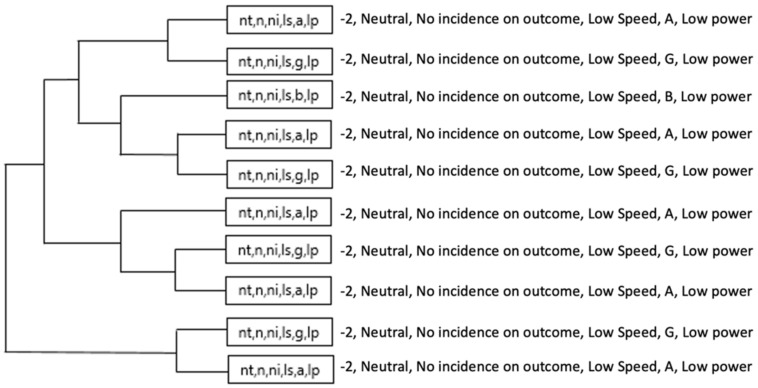
The most complex T-patterns detected in green team.

Figure 16 and Figure 17 illustrates a more complex dendrogram in Green team than Orange team. However, T-Patterns (categories) in Orange team were more dynamic by involving two types of scoring and two different roles (on, hr, ni, ls, a, lp), (on, hm, ni, ls, g, lp) and (tw, hr, ni, ls, a, lp), while in the Green team, only the acceleration and deceleration thresholds would vary (nt, n, ni, ls, a/g, lp).

Multimodal results 3. Frequency areas of strategic multimodal chains of Marro team and consignments

**Figure 18:**
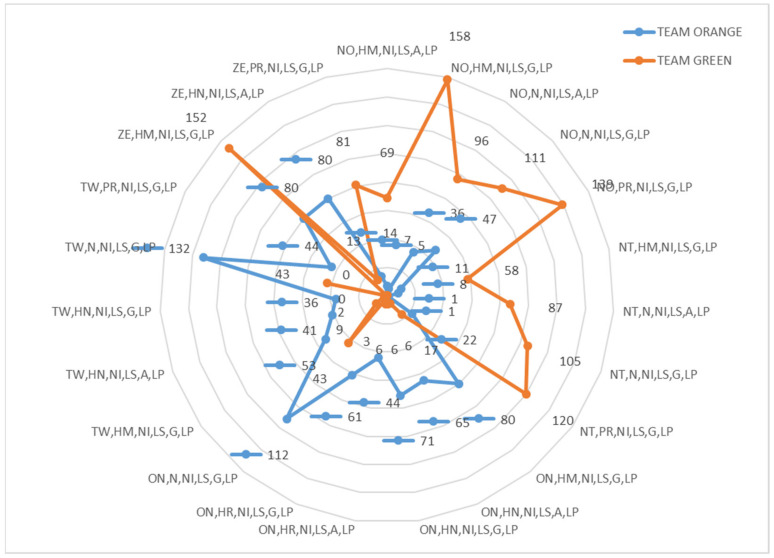
Frequency areas team Orange vs. team Green.

**Figure 19:**
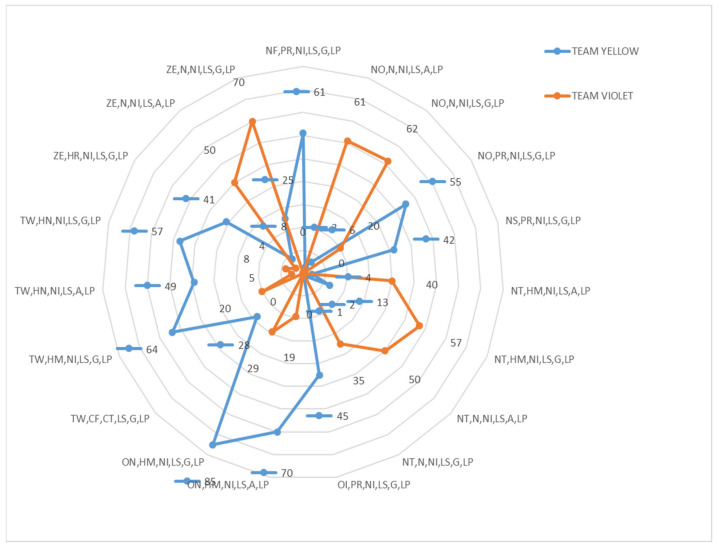
Frequency areas Yellow Team vs. Violet Team.

Figure 18 and Figure 19 illustrate the 360° multimodal strategies of the twenty-four participants in OG and YV matches. Combinations of Categorical variables with frequencies (≥35) or were considered, so combinations below these values were eliminated (*n* < 35). Also, values between +/−4 of the residual adjustments were used as an exclusion requirement. Therefore, all values (>4) and (<−4) were included.

The number of most repeated chains in the two matches was different. A higher frequency of repeated strings was found in the teams of the OG match than in the teams that faced each other in the YV match. OG Match (Orange Team *n* = 132 and 112; Green Team *n* = 80, 139, 152 and 158) and AL match (Yellow Team *n* = 70 and 80; Violet Team *n* = 61, 62 and 70).

In the Marro game the score was constantly modified (since the number of prisoners captured and saved changed without any established order). It was observed that the strategic chains that each team originated were activated in the face of unequal scores (Orange Team TW = +2; Green Team NO = −1 and NT = −2; Yellow Team ON = +1; Violet Team NO = −1).

The four teams (two matches) started their strategic chains from a different role, depending on whether their scoring was favourable or unfavourable. With a favourable score (TW or ON), the teams originated chains from the home or neutral role; while when the score was unfavourable (−2 or −1), the more numerous chains were activated from the prisoner (PR), neutral (N) and also home (HM) roles. Only one chain was identified associated with the conflict role (FC). The subroles corresponded to decision units with little energy relevance in the three variables: low speed (LS), low acceleration/deceleration (G = Acc/Dec 0–1 m/s); and low Met Pow (0–10 w/kg). In addition, in general with little motor relevance (neutral role, house, prisoner associated with decisions without incidence on the score).

The multi-modal chains most present in the different team were the following:

Match OG:

Orange Team: +2 TW, N, NI, LS, G, LP (*n* = 132); +2 TW, HM, NI, LS, G, LP (*n* = 112)

Green Team: Tie ZE, HM, NI, LS, G, LP (*n* = 152); −1 NO, HM, NI, LS, G, LP (*n* = 158); −1 NO, PR, NI, LS, G, LP (*n* =139); −2 NT, PR, NI, LS, G, LP (*n* = 80)

Match YV:

Yellow Team: +1 ON, HM, NI, LS, G, LP (*n* = 85); +1 ON, HM, NI, LS, A, LP (*n* = 70)

Violet Team: ZE, N, NI, LS, G, LP (*n* = 70); −1 NO, N, NI, LS, G, LP (*n* = 62); −1 NO, N, NI, LS, A, LP (*n* = 61)

**Orange Team vs. Green Team Duel**

**Yellow Team vs. Violet Team Duel**

## 4. Discussion

The aim of this research was to study of two matches of Marro game by using a 360° multimodal approach [2]. This multi-approach methodological premise made it possible to determine with enough specificity according to organic and decisional conducts (direct observation). The notion of ‘match’, as variable was novelty in research through TSGs.

### 4.1. First Results, Ready for a Multimodal Approach: The Case of the Marro Game

The multidimensional approach Marro game notes the importance of attending different dimensions in an intertwined way. Statistical analyses complement each other and allow us the 360° multimodal approach, considering roles, decisions or subroles and energy implications (speed, acceleration/deceleration and metabolic power). All of this was based on a key element of the internal logic of the Marro game: the scoring that was constantly changed. The most frequent result was a draw, followed by other scores such as ON (+1), NO (−1), TW (2) and NT (−2). In this game a team can reach tied, reaching tied again, or having a one-point advantage or it can even happen that a team manages to finish the game early, having reached the limit score, as happened in the match Yellow against the Violet team. This is another original feature of the game’s rule, which is confirmed by players. The teams of both matches experienced two different game adventures, where the scores during the game were different. The findings show the unpredictable and changing nature associated with a varied decision-making process, depending on the information uncertainty generated mainly by the opponents [4].

It has been observed that the two matches have followed different dynamics, so the Yellow and Violet teams of the YV match participated longer in the HN (Hunter) role than in the prisoner (PR) or neutral (N) roles. This superiority led to participation in motor actions of greater commitment or decisional risk. This does not mean that the HR role was not present in the OG role, since in the four team duels (YV and OG) the HN role was more present than the free HR role (Hare).

The multimodal vision facilitates the integrated approach of decisions with relationships. In this case, the conflict role played very little in both matches. Therefore, we can affirm that we are dealing with participants very respectful each other.

The most frequent (subrole) decisions (NI) do not have a direct impact on the score. We might think that this is a rather undynamic game. However, we may think that, above all, strategic interventions predominate, which serve to prepare the game’s high points: the capture of an opponent or the release of prisoners from our team.

The results from Speed, accelerations/decelerations and metabolic power complement the previous findings. Low values were observed in all three variables, which requires us to interweave the different classes of variables (scoring, role, subrole, speed, acceleration/deceleration and metabolic power). The data allowed us to estimate by an iterative procedure the time course of the speed and hence of the acceleration yielding an energy cost value such that when multiplied by the speed yields the actual metabolic power [33].

Metabolic power is not the panacea for team-sport analysis, but it is a very useful tool. By estimating the cost of acceleration in activity comprising perpetual changes in speed, it addresses a fundamental flaw of existing approaches. Consequently, it provides a more comprehensive—yet still incomplete—measure of intensity and volume for variable-speed locomotion, allowing for improved monitoring of training and competition loads and thus represents a step in the right direction in the quest to understand the demands of team sports. Metabolic power is not the panacea for team-sport analysis, but it is a very useful tool. By estimating the cost of acceleration in activity comprising perpetual changes in speed, it addresses a fundamental flaw of existing approaches. Consequently, it provides a more comprehensive—yet still incomplete—measure of intensity and volume for variable-speed locomotion, allowing for improved monitoring of training and competition loads and thus represents a step in the right direction in the quest to understand the demands of team sports. Metabolic power is not the panacea for team-sport analysis, but it is a very useful tool. By estimating the cost of acceleration in activity comprising perpetual changes in speed, it addresses a fundamental flaw of existing approaches. Consequently, it provides a more comprehensive—yet still incomplete—measure of intensity and volume for variable-speed locomotion, allowing for improved monitoring of training and competition loads and thus represents a step in the right direction in the quest to understand the demands of team sports.

Metabolic power is a very useful tool. By estimating the cost of acceleration in activity comprising perpetual changes in speed, it addresses a fundamental flaw of existing approaches. Consequently, it provides a more comprehensive—yet still incomplete—measure of intensity and volume for variable-speed locomotion, allowing for improved monitoring of training and competition loads and thus represents a step in the right direction in the quest to understand the demands of team sports [34].

Finally, the technology for data collection must be properly selected: when dealing with accelerations, any sample rate below 10 Hz is highly questionable. Additionally, signal filtering should be considered to smooth the accelerations/decelerations of the center of mass in sync with the stride frequency to reduce noise, without losing information [35].

Metabolic power is proposed [36] as a more accurate way of calculating the intensity because its calculation integrates both the speed and acceleration that the player demonstrates at each moment, instead of considering them separately.

### 4.2. Decision-Making Importance and Scoring: The Marro 360° Multimodal Approach

The classification trees establish a hierarchical order of the variables when it comes to finding the strategic specificity of both matches. The first variable Role (which integrates the different subroles) offers greater explanatory force. Secondly, the scoring intervenes, which confirms the importance of time in this game. In Marro, the player who comes out “last” has an advantage over the others (it is a relationship with time); the game is based on moving from one role to another (it is also a relationship with time) in a constant roles changes [1]. Finally, unlike sports, the scoring can move forward, backward or even a team can win before the agreed time has elapsed (again, the relationship with time is key).

The player must constantly detect, interpret and understand; he or she must pay attention to ongoing relationships and bring out new ones, in turn generating new organizations and creating a distribution of the game. The player is constantly in motion [37].

The strength of the decisional and relational temporality of the game justifies that the other three energy variables remain in the predictive background and appear in the last place in the hierarchy of the classification tree. This indicates that the decision takes precedence, and the energetic implication is at the service of the decision-making.

This type of observation has been less explored in sports competition [38] which is more interested in determining sports performance indicators. The paths followed by the matches obey the same internal processes that occur within the teams. However, it is up to the physical education teacher to increase a global, interpretative and reflexive approach, in order to fill his or her decisions with contextualised content, without excluding the fulfilment of current competences [39] TSGs.

With all this, the second methodological approach invites us to reveal multimodal strategic temporal regularities in the four teams, that is, in both matches.

### 4.3. In Search of T-Patterns through Strategic Multimodal Chains

Knowing the decisional anatomy of the teams or matches in any TSG requires addressing a range of connectivity that nests on roles and subroles to guide the study of game action, and thus build a comprehensive coding system; composed by criteria (roles) and categories (subroles), in line with observational studies, themselves considered mixed methods [32]. However, how to address the spontaneity of motor actions through TSGs? Linked to the mixed approach [40], in-depth analysis by specialists is required as a prerequisite for understanding the identification of the specificity of roles to play [41].

The TPA approach has revealed strategic regularities (T-Patterns) used by the four teams. In addition, the frequency areas provide a graphical (visual) aid to identify the most commonly used multi-modal chains by teams. These two complementary strategies suggest a more integrated view of the game dynamics, and allow us to advance a multimodal interpretation of the phenomenon being studied.

In the Marro game [42] is unavoidable to consider the temporal relationship of the players actions, understood as an unavoidable strategic indicator [2]. This approach distinguishes from other studies, more interested for sociological issues [43]; and thus, pay attention on increasing the understanding of the dynamics of matches and teams while playing. The real interest of this study has been ambitious, overcoming the isolated and fragmented vision of some data of the game: to reveal interconnected pieces of the two matches understood as a puzzle of interconnected relationships. How and when to send coded messages between partners, undecipherable by the rival threat?

The preference of actions aimed at capture over the rival in the role of Hunter should be understood as the visible part of the strategic iceberg of both matches. The findings from the TPA perspective reveal regularities of multimodal strategic time patterns reflected in the dendrograms of the four teams. The dendrograms reflect the selection of the most complex multimodal strategic chains with the highest impact on the scoring. The most relevant fact does not point towards the identification of regularities in the Hare role in both teams, but the favourable score that rested on the Yellow team (+2) could be due to the identification of the transition of roles [1] not identified in the Violet team. The same fact was detected in both matches, when the passive of the role in the Green team could facilitate the overcoming in the team’s score.

This is a game with an important decision-making process because the actions are fast-paced. In this sense, decision-making could be even more decisive than metabolic indicators. Speed, acceleration and deceleration, and metabolic power can be a consequence of the team’s plan. This action plan, as shown by the dendrograms and the frequency areas, devotes most of the game to motor interventions that do not directly affect the score. Players continually switch relationships to capture opponents or to save playmates. The game contains great originality in the rules, decisions and relationships and energy management.

These findings provide us a different view of the ludic context of TSGs, recognised as a scenario characterised by complexity [44] and playful spontaneity [45]. Within the apparent disorder that originates the superficial look of the game dynamics, there is a deeply strategic and temporal order [18,19,26] which suggests to take a distance from the identification of decision-making as random process (Limayem, and [46,47]).

Playing TSGs as Marro game can be beneficial for battling the problems of physical non-activity in modern western societies but also to serve as a link between the society and its citizens. By playing such traditional sports games, the players re-enact similar experiences of the local culture that were played by people of other generations in the past.

## 5. Conclusions

In this study, the responses of the players have been observed in depth according to the score in the form of strategic decisions, and how these affect the physical effort of the player.

The two groups were different because their strategic multimodal chains were unequal, despite certain similarities when analysing the two matches in different contexts. The physical effort has been similar in both matches. The findings confirm that Marro game could be categorized into intermittent efforts, and determinants in the high intensity score, but with a higher percentage of time in unemployment or with low physical activity.

We have observed that teams play similarly on certain scores, and it is intuitive that this may be determined by the risk assumed with a score for or against. But we must be cautious as no direct link is established between the risk to be assumed by the player and the analysis of the complexity of the action or the requirement of the opposing team.

Finally, no link is established between the modification of the score and the conditional response, since, in the Blue vs. Violet match, the score was more dynamic than in the Orange vs. Green match, but the energetic values were similar in both matches without evident differences in this sense.

## 6. Limitations and Future Prospects

One of the limitations of this study is the lack of a priori knowledge of the team’s strategic plans in order to evaluate the interventions in a richer context. On the other hand, addressing the emotional state of players would favour the understanding of the 360° vision of players’ motor conducts, in accordance with what is proposed by the science of motor action [1]. Finally, conducting interviews with players in their respective teams and rivals would help to understand the intentionality and significance of the findings around the participants’ strategic chains.

The original 360° multimodal vision provided by this line of research, almost unheard of in observational studies, proposes as future prospects to continue advancing in this type of study that facilitates the interpretation of the processes that occur in the development of a game, that is, to reveal the networks of interconnections that originate the implementation of the internal logic of any TSG.

As indicated by [33]:

“Each player is the agenda of a behaviour of motor interrelations to which he/she gives meaning within the confrontation of two teams. We are therefore at the heart of a group dynamic whose originality is to express itself through movement”

(2018, p. 91).

## Figures and Tables

**Table 1:** Thresholds set in the Speed variable according to gender.

	Code	Km/h (Women)	Km/h (Men)
Low	LS	−6	−7
Medium	MS	6–12	7–13
High	HS	12–16	13–18
Very high	VS	16–18	18–21
Maximum	XS	18–20	21–24
Extreme	EN	+20	+24

**Table 2:** Thresholds of the variable Acc/Dec.

Code in the Database	m/s
A	0 to −1
B	−1 to −2
C	−2 to −3
D	−3 to −4
E	−4 to −5
F	<−5
G	0 to 1
H	1 to 2
I	2 to 3
J	3 to 4
K	4 to 5
L	+5

**Table 3:** Thresholds of the Met Pow variable.

	Code	w/kg
Low	LP	0–10
Medium	MP	10–15
High	HP	15–20
Very high	VP	20–35
Maximum	XP	35–55
Extreme	EP	>55

**Table 4:** Transformation performed to convert sequential data to T-Data removing repeated events from original database (second by second) in order to be analyzed by THEME v.6.

Time Scale	6 Categories (7 Events)	Duration (Interval)	6 Categories (2 Events)
10 s	TW HN NI IS A IP	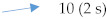	TW HN NI IS A IP
11 s	TW HN NI IS A IP	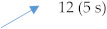	TW HN NI IS G IP
12 s	TW HN NI IS G IP		
13 s	TW HN NI IS G IP		
14 s	TW HN NI IS G IP		
15 s	TW HN NI IS G IP		
17 s	TW HN NI IS G IP		

Note: Following previous studies (Muñoz Arroyave et al., 2021) the order data corresponding to the 7 events, one per second (One Row = One Second) (from 10 to 17 s) were transformed into two intervals (One Row = Time Interval: 2″; 5″) (from 10 to 17 s) to be analyzed as T-Data.
